# Post COVID-19 Pandemic Increased Detection of Mycoplasma Pneumoniae in Adults Admitted to the Intensive Care

**DOI:** 10.3390/jcm13123443

**Published:** 2024-06-12

**Authors:** M. Goeijenbier, S. van der Bie, D. Souverein, D. Bolluyt, M. Nagel, S. P. Stoof, B. Vermin, J. Weenink, E. C. M. van Gorp, S. Euser, J. Kalpoe, M. A. van Houten, H. Endeman, D. Gommers, L. E. M. Haas, S. F. L. van Lelyveld

**Affiliations:** 1 Department of Intensive Care Medicine, Spaarne Gasthuis, 2035 RC Haarlem, The Netherlands; svanderbie@spaarnegasthuis.nl (S.v.d.B.); bvermin@spaarnegasthuis.nl (B.V.); jweenink@spaarnegasthuis.nl (J.W.); 2 Department of Intensive Care Medicine, Erasmus MC University Medical Centre, 3015 GD Rotterdam, The Netherlands; h.endeman@erasmusmc.nl (H.E.); d.gommers@erasmusmc.nl (D.G.); 3 Regional Public Health Laboratory Kennemerland, 2035 RC Haarlem, The Netherlands; d.souverein@streeklabhaarlem.nl (D.S.); s.euser@streeklabhaarlem.nl (S.E.); j.kalpoe@streeklabhaarlem.nl (J.K.); 4 Department of Internal Medicine, Spaarne Gasthuis, 2035 RC Haarlem, The Netherlandss.van.lelyveld@spaarnegasthuis.nl (S.F.L.v.L.); 5 Department of Intensive Care Medicine, Diakonessenhuis, 3582 KE Utrecht, The Netherlands; mnagel@diakhuis.nl (M.N.); lvlelyveld@diakhuis.nl (L.E.M.H.); 6 Department of Medical Microbiology and Immunology, Diakonessenhuis, 3582 KE Utrecht, The Netherlands; sstoof@diakhuis.nl; 7 Department of Viroscience, Erasmus MC University Medical Centre, 3015 GD Rotterdam, The Netherlands; e.vangorp@erasmusmc.nl; 8 Department of Paediatrics, Spaarne Gasthuis, 2035 RC Haarlem, The Netherlands; mavanhouten@spaarnegasthuis.nl

**Keywords:** *Mycoplasma pneumoniae*, community-acquired pneumoniae, outbreak report

## Abstract

**Background**: *Mycoplasma pneumoniae* (*M. pneumoniae*) infections can progress to severe respiratory complications, necessitating intensive care treatment. Recent post COVID-19 pandemic surges underscore the need for timely diagnosis, given potential diagnostic method limitations. **Methods:** A retrospective case series analysis was conducted on *M. pneumonia* PCR-positive patients admitted to two Dutch secondary hospitals’ ICUs between January 2023 and February 2024. Clinical presentations, treatments, outcomes, and mechanical ventilation data were assessed. **Results**: Seventeen ICU-admitted patients were identified, with a median age of 44 years, primarily due to hypoxia. Non-invasive ventilation was effective for most, while five required invasive mechanical ventilation. None of the patients required extracorporeal membrane oxygenation. No fatalities occurred. Post-PCR, treatment was adjusted to doxycycline or azithromycin; seven received steroid treatment. **Discussion**: Increased ICU admissions for *M. pneumoniae* infection were observed. Diverse clinical and radiological findings emphasize heightened clinical awareness. Early molecular diagnostics and tailored antibiotic regimens are crucial since beta-lactam antibiotics are ineffective. **Conclusion**: This study highlights the escalating challenge of severe *M. pneumoniae* infections in ICUs, necessitating a multifaceted approach involving accurate diagnostics, vigilant monitoring, and adaptable treatment strategies for optimal patient outcomes.

## 1. Background

*Mycoplasma pneumoniae* (*M. pneumoniae*) is linked to a broad range of diseases. Clinical presentation varies from mild respiratory complaints to a severe, life-threatening condition necessitating intensive care treatment or even invasive mechanical ventilation. While the need for intensive care treatment is relatively rare, periodic surges have been noted in the past, following a seasonal outbreak pattern [1]. Atypical manifestations of *M. pneumoniae* infections, including severe extrapulmonary symptoms such as cardiac arrhythmia and systemic disease, have also been documented [2].

During the winter season of 2023–2024, the Netherlands has experienced a significant increase in reported *M. pneumoniae* detections, aligning with a rise in *M. pneumoniae* incidence observed in the ESGMAC MAPS study, a global surveillance initiative monitoring *M. pneumonia* [3,4]. Considering other (preliminary) reports of increased *M. pneumoniae* prevalence in Western Europe with a concerning percentage potentially escalating to the intensive care unit (ICU), it is of great importance to implement molecular *M. pneumoniae* diagnostics within local diagnostic panels for respiratory infections [5]. Notably, *M. pneumoniae* infections often elude conventional diagnostic methods like sputum culture and Gram-staining, making Polymerase Chain Reaction (PCR) testing imperative for its detection [6]. Moreover, detecting *M. pneumoniae* at an early stage has significant implications for treatment, as first-line empiric treatment regimens for community-acquired pneumonia (CAP) typically do not incorporate antibiotics effective against *M. pneumoniae* [7].

After the COVID-19 pandemic, common respiratory pathogens in CAP have resurged [8], though their seasonality and prevalence could very well have shifted compared to pre-pandemic patterns, or is not yet known. Notably, Khoury et al. documented the only well-characterized pre-pandemic outbreak of *M. pneumoniae*, detailing the characteristics of patients requiring critical care [1]. Recent reports have highlighted a significant upsurge in *M. pneumoniae* infections [5,9]. This trend is concerning for several reasons. Firstly, empiric treatments for CAP often do not cover *M. pneumoniae*, potentially leading to inadequate initial therapy [7]. Secondly, the presence of co-circulating COVID-19 can cause delays in diagnosing *M. pneumoniae*, particularly if it is not included in the initial respiratory pathogen panel [10]. Lastly, in some parts of the world, there is a high incidence of marcolide resistance complicating treatment protocols and patient outcomes [7,11,12].

This sudden uprise in *M. pneumoniae*-associated pneumonia underscores a critical need for a meticulous diagnostic and treatment approach. This is emphasized even more by the high percentage of patients with a positive *M. pneumoniae* PCR in a short time frame who were admitted to the ICU in our hospitals. In this retrospective study, we focus on clinical outcomes and data on (mechanical) ventilation in *M. pneumoniae* patients admitted to intensive care in two large teaching hospitals in the Netherlands.

## 2. Methods

We performed a retrospective analysis of the case series of patients with a positive *M. pneumoniae* PCR admitted to the ICU between 1 January 2023 and 14 February 2024 in Spaarne Gasthuis Haarlem/Hoofddorp (560 beds and 54,000 annual visits to the emergency department) and Diakonessenhuis Utrecht (553 beds and 27,000 annual ED visits), both in the Netherlands. Hospital admission related to *M. pneumoniae* was defined as the detection of *M. pneumoniae* in a respiratory sample (naso- and/or oropharyngeal (NP/OP) swab, sputum, or bronchoalveolar lavage (BAL) fluid) of an admitted patient using PCR-based diagnostic tests. For Spaarne Gasthuis, respiratory pathogen detection (including *M. pneumonia*) was evaluated using multiplex ligation-dependent probe amplification (MLPA) PCR [13]. For Diakonessenhuis, respiratory samples were analyzed for the presence of *M. pneumoniae* using in-house real-time PCR. Searches were performed in the databases of the Regional Public Health Laboratory Kennemerland (Spaarne Gasthuis) and the department of medical microbiology of the Diakonessenhuis Utrecht. All the patients with the detection of *M. pneumoniae* in a respiratory sample by PCR admitted to the ICU were included.

In addition, epidemiological data for Spaarne Gasthuis regarding the absolute number of performed *M. pneumoniae* PCR tests, the number of positive *M. pneumoniae* PCR tests, and the total number of hospital and ICU admissions were electronically extracted from the database of the Regional Public Health Laboratory Kennemerland. For this purpose, hospital admission related to *M. pneumoniae* was defined as the detection of *M. pneumonia* in a respiratory sample using PCR-based diagnostic tests up to 2 days before or during admission.

Clinical data collection was performed by extracting information from the electronic patient records, such as demographic data, medical history, clinical symptoms, laboratory and imaging results, the parameters of mechanical ventilation, the duration of ventilation, the length of hospital stay, treatment, and mortality.

Statistical analysis consisted solely of descriptive statistics. Categorical variables were displayed as percentages. Continuous variables were displayed with a mean (+/− standard deviation) for normally distributed variables and median (interquartile range) in case of a non-normally distributed variable. All analyses were performed with R and Graph Pad Prism 10.0 (version 10.1.0 (264)).

This study was approved by the Institutional Review Board of the Spaarne Gasthuis (2023.0136) and the hospital’s local review committee of Diakonessenhuis Utrecht (23-068). Procedures were followed in accordance with the ethical standards of the responsible committee on human experimentation (institutional or regional) and with the Helsinki Declaration of 1975.

## 3. Results

**Figure 1:**
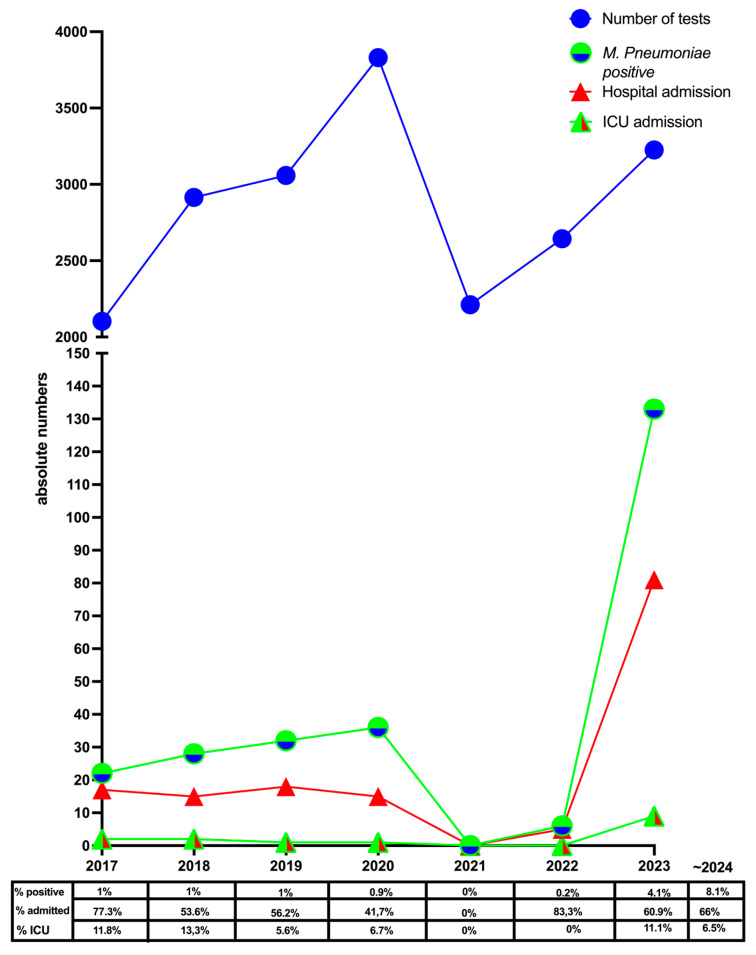
Absolute number of *M. pneumoniae* PCR tests performed, positive *M. pneumoniae* PCR, and hospital and ICU admissions in Spaarne Gasthuis with a positive *M. pneumoniae* PCR is plotted.

Between 1 January 2023 and 14 February 2024, 141 (3.7%) patients with *M. pneumonia* were detected out of 3809 *M. pneumonia* PCR tests performed in Spaarne Gasthuis. Of these, 112 (79.4%) patients were admitted to the hospital and 11 (9.8%) to the ICU (Figure 1). In Diakonessenhuis, 488 *M. pneumonia* PCR tests were performed, of which 81 (16.6%) were positive. Fifty-eight (71.6%) patients were admitted to the hospital, of which five (8.6%) patients were admitted to the ICU. One additional patient was transferred to the ICU from another hospital. In general, the percentage of positive *M. pneumoniae* PCR was clearly increased in the Spaarne Gasthuis during the period 2023–2024 as compared to previous years, as can be seen in Figure 1. Although the number of hospital and ICU admissions increased due to the higher number of *M. pneumoniae* detection, the percentage of admissions did not significantly increase (*p* = 0.421).

The figure table shows the percentage of hospital and ICU admissions of the total positive tests within Spaarne Gasthuis before the COVID-19 pandemic, during the pandemic, and in 2023.

Table 1 summarizes the patient characteristics of the 17 patients admitted to both ICU departments. All the patients were admitted to ICU during the period September 2023–January 2024. Briefly, the median age of these patients was 44 years (IQR 29 to 62) and 18% was female (82% male). Eight (53%) patients had underlying comorbidities and six (40%) patients had a history of smoking. Fourteen patients were admitted to the ICU because of hypoxia.

The median duration of hospital admission was 10 days (IQR 7–14), of which 5 (IQR 2–7) were spent in the ICU. Most patients benefitted from non-invasive ventilation support including nasal high-flow therapy (NHFT) (70%). However, invasive mechanical ventilation (IMV) was necessary in five (30%) of the patients, and the mean duration of IMV was 6 days (IQR 4–9). When on IMV, in 3 out of 5 cases P/F ratio was below 200 mmHg and proning was initiated. None of the patients warranted treatment with (venous-venous) extra corporal membrane oxygenation.

None of the patients with *M. pneumoniae* infection died during admission. In all the patients’ initial empiric treatment, either started on ICU admission or already previously on hospital admission, was a beta-lactam antibiotic (third-generation cephalosporin) with or without fluoroquinolones treatment, a macrolide or doxycycline. When PCR on *M. pneumoniae* turned out to be positive, treatment with doxycycline or azithromycin was started at the clinician’s discretion for a median of 14 [7–14 IQR] and 5 [3.5–5] IQR days, respectively, while in three patients both doxycycline and azithromycin were started alternately. Steroid treatment was started in 47% of the patients and treatment strategies differed (listed in Table 2).

Because of the relatively small number of patients in this brief report and the large diversity in clinical signs, symptoms, and outcomes, we summarized the signs and symptoms at presentation, course of disease, main reason for ICU admission, and main radiology findings in Table 2. Radiology findings differed from lobar consolidations to bilateral pathology with pleural effusion only in a minority of cases. In six patients, there were co-infections with/co-detections of other micro-organisms (e.g., *S. pneumonia; B. pertussis*). In these cases, the relative contribution of *M. pneumonia* to the clinical condition responsible for ICU admission is uncertain; however, in all the cases, clinicians judged it necessary to start antibiotic treatment for *M. pneumonia* as well. In Table 2, we also listed the ROX index, if available, for intubation after high-flow nasal canula treatment, (ROX Index = SpO₂/FiO₂*, %/Respiratory rate, breaths/min) to predict the probability of HFNC failure [14]. Although in a small sample, the patients with successful HFNC treatment, meaning without the necessity to start IMV, all had ROX scores above 3.85.

## 4. Discussion

In this article, we summarize clinical data and outcomes from 17 patients admitted to the ICU due to *M. pneumoniae* infection in a short time frame (September 2023–January 2024). Considering the pandemic potential of *M. pneumoniae*, we believe it is important to share as much clinical data as possible in the early stage of the notification of increased infections. Although the number of infections seems to decrease in the Netherlands, ICU admission rates are expected to rise in multiple parts of the world.

Although in a relatively small cohort, we observed a broad range of clinical signs and symptoms, radiology findings exceeding the classical “tree-in-bud” sign and a relatively high percentage of ICU admissions. Also, the number of young adults seemed surprisingly high. In line with previous reports from a *M. pneumoniae* outbreak in 2016, the new onset of cardiac arrhythmias during *M. pneumoniae* infection could also be a reason for medium, high, or even ICU admission, as was the case in two of our patients [1]. Furthermore, in patients admitted to the ICU not in need of IMV, high-flow oxygen treatment by nasal cannula seemed effective.

Of great interest is the discussion of potential explanations regarding the recent upsurge of *M. pneumoniae* in Europe, and the question if global numbers also will increase, leading to higher ICU admission rates.

In a recent global study by Sauteur et al., the correlation between the implementation of Non-Pharmaceutical Interventions (NPIs) for COVID-19 and a significant reduction in *M. pneumoniae* infections was observed. The study postulates that certain NPIs retained post-COVID-19, such as improved hand hygiene and respiratory etiquette, may contribute to limiting *M. pneumoniae* transmission. This correlation, elucidated from diverse geographical locations, underscores the potential impact of external factors on *M. pneumoniae* epidemiology. However, during this period of NPIs herd immunity might also be affected, potentially in combination with certain *M. pneumoniae*-specific characteristics such as slow generation time, long incubation period, and relatively low transmission rate resulting in a delayed re-emergence of *M. pneumoniae* potentially with a higher number of infections [3,8].

Despite variations observed across multiple studies, previous research conducted prior to the implementation of NPIs for COVID-19 exhibits data that aligns closely with our findings, including extrapulmonary manifestations in severe *M. pneumoniae* infections like cardiac arrhythmias [10,11]. Alterations in clinical characteristics may suggest a shift in the virulence and pathogenicity of the bacterium.

In the Netherlands, and other parts of Europe, current empiric first-line treatment regimens for CAP mainly consist of beta-lactam antibiotics such as amoxicillin or cephalosporins, which are ineffective against *M. pneumoniae*. It is therefore imperative that clinicians have a high index of suspicion for atypical micro-organisms causing CAP such as *M. pneumoniae*, particularly those with atypical clinical presentations or insufficient response to empiric antibiotic treatment for CAP [2,5,10,12]. The addition of quinolone treatment, which is advised in the most severe cases of CAP, does not always cover *M. pneumoniae* in general, as is the case for ciprofloxacin. Furthermore, the implementation of macrolide treatment before a positive PCR test does not guarantee success in certain areas of the world since macrolide resistance has been reported, especially in Asia [11]. This underscores the urgency for overall awareness of diagnostic and therapeutic strategies employed for *Mycoplasma pneumoniae*-induced pneumonia. Additionally, this study highlights a disparity in testing approaches. Whereas *M. pneumoniae* is routinely included in supplementary respiratory PCR testing protocols at Spaarne Gasthuis, Diakonessenhuis restricts testing to instances of increased suspicion. Detection rates were below 5% at Spaarne Gasthuis as compared to over 16% at Diakonessenhuis. Emphasizing the present resurgence and the potential severity of the condition, prompt initiation of *M. pneumoniae* testing is warranted.

Furthermore, the addition of corticosteroids was not homogenous in this small retrospective case series. Although, based on recent RCTs suggested as add-on treatment in severe CAP [15], the corticosteroid effect on severe *M. pneumoniae* infection in adults has not been studied. Because of the small number and different steroid regimens used, it is not feasible to compare outcomes within this study based on steroid treatment.

The strength of our study is the relatively large number of patients admitted to the ICU because of *M. pneumonia* infection in a short recent period in combination with a thorough presentation of patient characteristics and symptoms. However, this study has several limitations. First, the retrospective observational nature of the study with its limitations. Second, diagnostics relied on molecular detection by PCR which has a known limitation for its inability to differentiate between asymptomatic colonization or symptomatic infection. The distinction between colonization and infection with *M. pneumoniae* is particularly challenging in NP/OP-swabs, as carriership can also occur in that context. For example, high asymptomatic *M. pneumoniae* carriage rates have been described in children [16] Especially the contribution of *M. pneumoniae* to the clinical condition of patients in our cohort with the detection of other pathogens is not certain. However, in these cases, clinicians judged it necessary to cover *M. pneumoniae* in the antibiotic regimen as well. Finally, whether non-invasive ventilation is used in the ICU or another department may differ between countries and even hospitals. Therefore, in other healthcare settings, not all patients in our cohort might have been admitted to the ICU. In spite of these shortcomings, we believe our data provide new and valuable insights into patients (admitted to the ICU) with *M. pneumonia* infection and call for increased awareness in global critical care, especially when circulating SARS-CoV-2 strains, like Omicron, no longer cause severe disease in the majority of cases but remain the main pathogen tested for in suspected (severe) acute respiratory infections. For instance, Freund et al. describe a statistically significant delay, but potentially also clinically significant, in a second diagnosis after a positive SARS-CoV-2 PCR especially in those patients with severe cough, dyspnea, or a low oxygen saturation [10]. Lastly, in the post-pandemic era, there is a high need for prospective cohort studies in severe acute respiratory infections in multiple geographical locations redefining seasonality, severity, and prevalence of known, or unknown, respiratory pathogens.

## 5. Conclusions

We presented a case series of 17 patients admitted to the ICU because of *M. pneumoniae* infection, with a significant number of young adults, 33% needed IMV, and a high diversity in clinical presentation. All were discharged alive from the hospital. As *M. pneumoniae* infections resurge, our findings stress the importance of a comprehensive diagnostic approach, including targeted molecular diagnostics, and a reconsideration of treatment strategies to effectively address the challenges posed by this evolving respiratory pathogen.

## Figures and Tables

**Table 1:** Characteristics of Mycoplasma pneumoniae-infected patients admitted to the intensive care unit.

	Total
**Age (years) [median (IQR)**	44.0 (29.0 to 62.0)
**Sex (female)**	3 (17.6)
**BMI (kg/m^2^) median (IQR)**	26.3 (22.4 to 28.5)
**Cardiovascular disease**	4 (23.5)
**Preexisting respiratory disease (COPD, etc.)**	2 (11.8)
**Chronic renal insufficiency**	1 (5.9)
**Neurological disorder**	1 (5.9)
**Malignancy**	1 (5.9)
**Diabetes Mellitus**	2 (11.8)
**Auto-immune disease**	2 (11.8)
**Immune suppression**	1 (5.9)
**Smoking (current or previous)**	6 (35.3)
**Harddrugs use**	2 (11.8)
**Hypoxia as the reason for ICU admission**	14 (82.4)
**Hemodynamic instability as a reason for ICU admission**	3 (17.6)
**APACHE score on admission [median (IQR)**	45.0 (37.0 to 52.0)
**SAPS II on admission median (IQR)**	21.0 (18.0 to 24.0)
**Leukocyte on admission (×10^9^/L) median (IQR)**	11.3 (9.2 to 17.1)
**CRP (mg/L) on presentation median (IQR)**	169 (102 to 304)
**Non-invasive ventilation including high flow**	13 (76.5)
Duration of non-invasive ventilation (hours/days) median (IQR) *	72 (24–108) 3 (1–4.5)
**Invasive mechanical ventilation (IMV)**	5 (29.4)
**Duration of invasive mechanical ventilation (IMV) (days) median (IQR) ^†^**	6.0 (4.0 to 9.0)
**Highest FIO_2_ (%) median (IQR)]**	75.0 (60.0 to 85.0)
**Lowest PaO_2_ (kPa) at admission under maximal FIO_2_ median (IQR)**	9.0 (8.2 to 10.1)
**Lowest PF ratio (mmHg) in mechanically ventilated patients median (IQR) ^‡^**	85.0 (77 to 110)
**Highest PaCO_2_ (kPa) median (IQR)**	6.0 (5.2 to 6.4)
**SpO2 at admission ICU with oxygen suppletion median (IQR)**	93.0 (92.0 to 95.0)
**FIO2 at admission ICU median (IQR)**	70.0 (60.0 to 90.0)
**ROX-score (pulse oximetry/FIO21) at admission ICU median (IQR)**	5.6 (4.6 to 13.1)
**Highest PEEP (cmH_2_O) median (IQR) ^§^**	14.0 (12.0 to 14.0)
**Highest driving pressure (cmH2O) median (IQR) ^¶^**	15.0 (13.8 to 16.8)
**Lowest pH median (IQR)**	7.4 (7.3 to 7.4)
**Proning**	3 (17.6)
**AKI during ICU stay**	2 (11.8)
**Steroid treatment**	7 (41.2)
Duration of doxycycline treatment (days) median (IQR) **	14.0 (7.0 to 14.0)
**Duration of azithromycin treatment (days) median (IQR) ^††^**	5.0 (3.5 to 5.0)
**Duration of hospital admission (days) median (IQR)**	10.0 (7.0 to 14.0)
**Duration of intensive care admission (days) median (IQR)**	5.0 (2.0 to 7.0)

Data were presented as numbers and percentages or indicated otherwise. IQR: interquartile range. Data were available for n patients: *: 13 patients, ^†^: 5 patients, ^‡^: 7 patients, ^§^: 5 patients, ^¶^: 4 patients, **: 14 patients, ^††^: 6 patients. None received renal replacement therapy. Mortality was 0% in this retrospective cohort.

**Table 2:** In depth case description, mechanical ventilation (both invasive and non-invasive data) and radiology findings.

Age/Sex	Comorbidity	Co-Detections	ICU Admission	Radiology	SOFA/APACHE/SAPS II	Duration ICU Stay	Summary
62/M	Heroin abuse	*Stenotrophomonas maltophilia* (sputum culture)	Hypoxia	Bilateral consolidation, multilobar infiltrates, pleural effusion	4/82/29	14 days	IMV: yes, duration 13 days Proning: yes
66/M	nsclc in active treatment	Rhinovirus (PCR) *Streptococcus pneumonia* (sputum culture)	Hemodynamic instability with new onset SVT	Interlobar septa thickening, pleural effusion, ground glass opacities	2/52/24	1 day	IMV: no NIMV: no Hemodynamic instability (new onset arrhythmia)
72/M	Diabetes mellitus type 2, smoking	None	Hypoxia	Bilateral consolidations	4/59/36	4 days	IMV: no NIMV: high-flow nasal cannula ROX at admission 3.61
18/M	Wilms tumor at age 1	None	Hypoxia	Ground glass, bilateral consolidations, tree in bud	4/39/17	7 days	IMV: yes, duration 6 days Proning: yes (non-responder) NIMV: high-flow nasal cannula ROX at admission: 2.8
50/F	None	*Bordetella pertussis* (PCR NP/OP swab)	Hemodynamic instability with new onset SVT	Consolidation right lower lobe	2/39/36	1 day	IMV: no NIMV: no Hemodynamic instability (new onset arrhythmia)
29/M	West syndrome with tetraparesis	None	Hypoxia	Retro cardiac consolidation	4/26/12	2 days	IMV: no NIMV: yes, 48 h of high-flow nasal cannula ROX at admission: 5.47
60/M	None	None	Hypoxia	Left peri hilar consolidation	1/49/18	5 days	IMV: no NIMV: yes, 108 h of high-flow nasal cannula ROX at admission: 3.26
48/M	Smoking	Adenovirus (PCR NP/OP swab)	Upper airway obstruction	No pulmonary pathology; soft tissue/lymphnode swollen, no abscess, no signs of sinusitis	5/25/22	3 days	IMV: yes, upper airway obstruction. Duration 48 h
43/M	None	Rhinovirus (PCR NP/OP swab)	Hypoxia	Consolidations right lower lobe and midlle lobe and left lower lobe	1/38/18	6 days	IMV: no NIMV: 5 days of high-flow nasal cannula ROX at admission: 7.46
77/M	Organizing pneumonia	None	Hypoxia	Right lower middle and upper lobe new consolidations, pleural effusion right	2/79/24	6 days	IMV: no NIMV: 5 days of high-flow nasal cannula ROX at admission: 5.17
27/F	None	None	Hypoxia	Consolidation left lower lobe	2/25/9	5 days	IMV: no NIMV: 4 days of high-flow nasal cannula ROX at admission: 8.61
26/M	None	None	Hypoxia	Reticonodular consolidations right lower lobe	4/37/22	7 days	IMV: yes, duration 4 days NIMV: yes
36/M	None	None	Hypoxia	Consolidation righ lower lobe and peribronchial cuffing	4/41/17	11 days	IMV: no NIMV: high-flow nasal cannula ROX at admission: -
44/M	Coeliac disease	None	Hypoxia	Bilateral consolidations	3/45/20	19 days	IMV: yes, duration 9 days Proning: yes NIMV: yes
36/M	None	None	Hypoxia	Reticonodular consolidations in the lower lobes	4/46/21	5 days	IMV: no NIMV: high-flow nasal cannula
29/F	Colitis Ulcerosa	None	Hypoxia	Bilateral patchy consolidations	3/51/21	2 days	IMV: no NIMV: high-flow nasal cannula
65/M	Heart failure, OSAS, chronic renal insufficiency, diabetes mellitus type 2	SARS-CoV-2 (PCR NP/OP swab), *Streptococcus pneumonia* (pleural fluid and blood culture)	Hemodynamic instability	Consolidation left lower lobe with pleural effusion	5/79/37	1 day	IMV: no NIMV: no

## Data Availability

Data are available upon request.
